# Spectinamides are effective partner agents for the treatment of tuberculosis in multiple mouse infection models

**DOI:** 10.1093/jac/dkw467

**Published:** 2016-12-13

**Authors:** Gregory T. Robertson, Michael S. Scherman, David F. Bruhn, Jiuyu Liu, Courtney Hastings, Michael R. McNeil, Michelle M. Butler, Terry L. Bowlin, Robin B. Lee, Richard E. Lee, Anne J. Lenaerts

**Affiliations:** 1 Mycobacterial Research Laboratories, Department of Microbiology, Immunology and Pathology, Colorado State University, Fort Collins, CO 80523, USA; 2 Department of Chemical Biology and Therapeutics, St Jude Children’s Research Hospital, Memphis, TN 28105, USA; 3 Microbiotix, Inc., Worcester, MA 01605, USA

## Abstract

**Objectives:** New drug regimens employing combinations of existing and experimental antimicrobial agents are needed to shorten treatment of tuberculosis (TB) in humans. The spectinamides are narrow-spectrum semisynthetic analogues of spectinomycin, modified to avoid intrinsic efflux by *Mycobacterium tuberculosis*. Spectinamides, including lead 1599, have been previously shown to exhibit a promising therapeutic profile in mice as single agents. Here we explore the *in vivo* activity of lead spectinamides when combined with other agents.

**Methods:** The efficacy of 1599 or 1810 was tested in combination in three increasingly advanced TB mouse models. Mice were infected by aerosol and allowed to establish acute or chronic infection, followed by treatment (≤4 weeks) with the spectinamides alone or in two- and three-drug combination regimens with existing and novel therapeutic agents. Bacteria were enumerated from lungs by plating for cfu.

**Results:** Herein we show the following: (i) 1599 exhibits additive or synergistic activity with most of the first-line agents; (ii) 1599 in combination with rifampicin and pyrazinamide or with bedaquiline and pyrazinamide promotes significantly improved efficacy in the high-dose aerosol model; (iii) 1599 enhances efficacy of rifampicin or pyrazinamide in chronically infected BALB/c mice; and (iv) 1599 is synergistic when administered in combination with rifampicin and pyrazinamide in the C3HeB/FeJ mouse model showing caseous necrotic pulmonary lesions.

**Conclusions:** Spectinamides were effective partner agents for multiple anti-TB agents including bedaquiline, rifampicin and pyrazinamide. None of these *in vivo* synergistic interactions was predicted from *in vitro* MIC chequerboard assays. These data support further development of the spectinamides as combination partners with existing and experimental anti-TB agents.

## Introduction

New drug regimens based on combinations of existing and experimental classes of antimicrobial agents are desperately needed to shorten and simplify treatment of TB infection caused by MDR or XDR *Mycobacterium tuberculosis*. Current TB research has therefore focused on the discovery and development of novel agents that complement and extend the efficacy of both first- and second-line TB agents, as well as TB agents under clinical development.^1^^,^^2^

The spectinamides are novel, semisynthetic antibiotics with a narrow spectrum of activity against *M. tuberculosis* and closely related mycobacterial species, lack cross-resistance to existing TB drugs and are active under hypoxic conditions.^3^ The molecular basis for this improved activity has been attributed to selective ribosomal inhibition, combined with synthetic chemical modifications that allowed evasion of the Rv1258c efflux pump. Efflux by Rv1258c is responsible for the poor intrinsic activity of the parent compound, spectinomycin (Figure S1, available as Supplementary data at *JAC* Online).^3^ Lead spectinamides exhibit a long post-antibiotic effect and a short serum half-life owing to extensive renal elimination, and are efficacious in both acute and chronic mouse models of TB.^3^ Poor oral bioavailability in this series has limited its usage to an injectable form so far; however, owing to their hydrophilic nature, the spectinamides have proven highly effective when administered via inhalation in mouse models.^3^ We recently explored the potential *in vivo* benefit for combination of the spectinamides with antibiotics not typically used to treat TB. In this published study, 1599 did not act as a general efflux pump inhibitor and synergy was only observed with non-conventional TB agents that also inhibited protein synthesis.^4^ Building from these studies, herein we investigated the efficacy of 1599 in combination with conventional anti-TB agents, including agents under late-stage clinical development. Synergistic interactions were assessed by conventional *in vitro* chequerboard assays and also through a series of short-term (≤4 weeks) acute and chronic murine infection models of TB, including C3HeB/FeJ mice exhibiting advanced, hypoxic, caseating granulomas.^5^^,^^6^ The resulting data revealed appreciable *in vivo* synergy between lead spectinamides and existing therapeutics, as well as compounds in late-stage clinical development that were not otherwise predicted by *in vitro* MIC chequerboard testing. Further development of this series and exploration of the mechanisms of synergy observed in these studies, using longer treatment durations in hard-to-treat caseous necrotic pulmonary lesions, may lead to new treatment options for human TB.

## Materials and methods

### Bacteria

*M. tuberculosis* H37Rv was used for *in vitro* chequerboard studies. *M. tuberculosis* Erdman strain^7^ or Erdman transformed with pFCA-LuxAB^8^ (Erdman-Lux) was used for drug evaluations in mice. The luciferase signal expressed from Erdman-Lux provided an early estimate of bacterial burden in lung and spleen homogenates of mice, with no appreciable decrease in virulence in conventional mouse infection models of TB. Excluding vector-encoded kanamycin resistance, Erdman and Erdman-Lux have shown equivalent susceptibility to spectinamides and standard TB drugs *in vitro*. Bacteria were prepared for infection as described previously.^5^

### Drug compounds

1599 and 1810 were synthesized as described (Figure S1).^3^ Isoniazid, rifampicin, pyrazinamide, ethambutol, levofloxacin, kanamycin, amikacin, ethionamide, ofloxacin, cycloserine, rifapentine and clofazimine were purchased from Sigma-Aldrich. Linezolid was purchased from 21CEC. Moxifloxacin was obtained from Bayer. PA-824 (Pretomanid) was obtained from the Global Alliance for TB Drug Development. Bedaquiline (Sirturo) was provided by Johnson and Johnson. SQ109 was synthesized as described.^9^

### Drug preparation for in vivo models

Spectinamides were formulated in Plasma-Lyte A (pH 7.4) diluted 5:1 with sterile water. Rifampicin, isoniazid, pyrazinamide, ethambutol, levofloxacin and moxifloxacin were ground by mortar and pestle and dissolved in sterile water. Linezolid was dissolved in 5% (w/v) PEG-200 (Sigma-Aldrich) followed by a 20-fold dilution with 0.5% (w/v) methyl cellulose (Fisher Scientific). PA-824 was formulated in 10% (w/v) 2-hydroxypropyl-β-cyclodextrin (2-HPCD) (Sigma-Aldrich) and 10% (w/v) lecithin (Sigma-Aldrich) as described.^7^ Bedaquiline was dissolved in 20% (w/v) 2-HPCD and formulated as described previously.^10^^,^^11^

### Ethics

All animal studies were performed at Colorado State University in a certified bio-safety level III animal facility in accordance with the guidelines of the Colorado State University Institutional Animal Care and Use Committee (reference number 13-4477A).

### Infection of mice

Aerosol infection of mice with *M. tuberculosis* Erdman or Erdman-Lux employed a Glas-Col inhalation exposure system as described.^12^

### Drug delivery and dose

1599 or 1810 was administered in a 0.2 mL volume by subcutaneous injection at 200 mg/kg. Isoniazid, rifampicin, pyrazinamide, ethambutol, linezolid, levofloxacin, moxifloxacin, PA-824 and bedaquiline were administered by oral gavage in a 0.2 mL volume at 25, 10, 150, 100, 100, 100, 100, 50 and 25 mg/kg, respectively. For combination studies, mice were first administered 1599 or 1810 by subcutaneous injection followed by administration of the combination agent(s) by oral gavage. In cases where mice were administered two or more oral drugs, these were combined to limit the total volume of oral administration to 0.2 mL. All treatments were given once daily, 5 days a week (Monday to Friday).

### BALB/c mouse high-dose aerosol infection model

Female pathogen-free BALB/c mice (6–8 weeks old; Charles River) were exposed to high-dose aerosol of *M. tuberculosis* Erdman or Erdman-Lux from broth culture (optical density at 600 nm of ∼0.8) to achieve deposition of ∼3.5–4.7 log_10_ cfu in the lungs of each mouse.^13^^,^^14^ Treatment was initiated on day 12 post-aerosol and continued for 3–4 weeks. Groups of five or six mice were sacrificed on day 12, prior to treatment initiation, and 3 days following the last day of treatment, to determine the bacterial loads in the lungs of mice. Mice treated with bedaquiline were sacrificed 5 days following the last day of treatment to allow drug clearance.

### BALB/c mouse chronic infection model

Female pathogen-free BALB/c mice (6–8 weeks old; Charles River) were exposed to a low-dose aerosol of *M. tuberculosis* Erdman-Lux using 2 × 10^6^ cfu/mL to achieve 50–100 cfu in the lungs of each mouse. Treatment was initiated on day 41 post-aerosol and continued for 4 weeks. Groups of three to six mice were sacrificed on day 41, prior to treatment initiation, and 3 days following the last day of treatment, to determine the bacterial loads in the lungs of mice.

### C3HeB/FeJ mouse chronic infection model

Female C3HeB/FeJ mice (6–8 weeks old; Jackson Laboratories) were exposed to a low-dose aerosol of *M. tuberculosis* Erdman using 1.5 × 10^6^ cfu/mL to achieve ∼50–75 cfu in the lungs of each mouse.^5^ Treatment was initiated on day 56 at the time when necrotic lesions had already developed^6^ and continued for 4 weeks. Groups of four or seven mice were sacrificed on day 56, prior to treatment initiation, and 3 days following the last day of treatment to determine the bacterial loads in the lungs of mice.

### Histological analysis

Lung tissues were collected and processed as described.^6^^,^^15^

### Enumeration of cfu from lungs

The number of viable organisms was determined by serial dilutions of homogenates (Glas-Col, Inc.) from whole lungs (C3HeB/FeJ) or left lung lobes (BALB/c) plated on 7H11-OADC. Lungs from bedaquiline-treated animals were homogenized in phosphate-buffered saline plus 10% (w/v) bovine serum albumin and plated on 7H11-OADC agar plates containing 0.4% (w/v) activated charcoal to prevent drug carry-over.^16^ Colonies were enumerated after at least 21 days of incubation at 37 °C. *In vivo* synergy, additivity or antagonism when evaluating the combined effects of two or more drugs was defined as follows: greater than the combined efficacies of each drug alone, synergy; no greater than the combined efficacies of each drug alone, additivity; and less than the efficacies of each drug alone, antagonism.

### Statistical analysis

Data were evaluated by a one-way analysis of variance followed by a multiple-comparison analysis of variance by a one-way Tukey test (SAS software program). Differences were considered signiﬁcant at the 95% level of conﬁdence.

### In vitro drug combination chequerboard assays

*In vitro* synergy was assessed using *M. tuberculosis* strain H37Rv, which was cultured as described previously.^17^ Fractional inhibitory concentration index (FICI) scores were calculated as described.^18^ FICIs were reported as the range of two independent experiments and were interpreted as follows: synergy, ≤0.5; indifference, >0.5–4.0; antagonism, >4.0.^18^

## Results

### Bactericidal activity of 1599 with anti-MDR TB drugs in BALB/c mice

**Figure 1:**
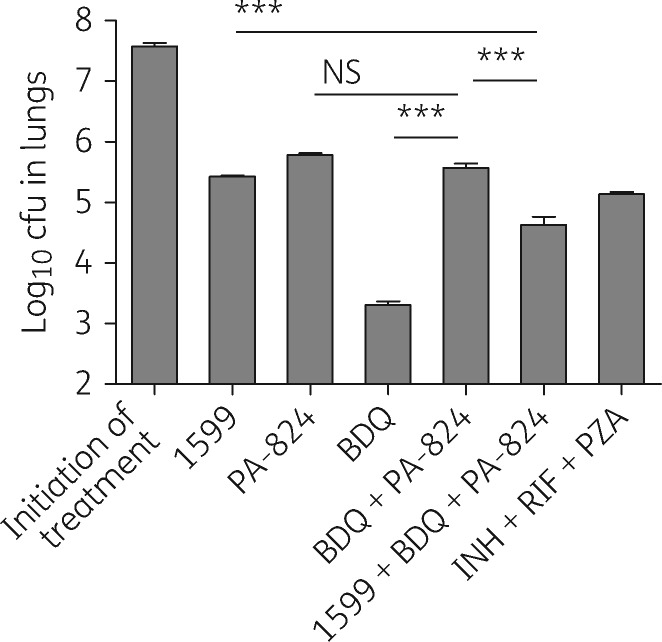
Evaluation of 1599 in combination with novel anti-TB agents under development in BALB/c mice following high-dose aerosol infection with *M. tuberculosis* Erdman-Lux. Bars represent mean log_10_ cfu values ±SEM from the lungs of individual BALB/c mice. Mice were treated for 3 weeks with the following drugs, alone or in various combinations: 1599 at 200 mg/kg by subcutaneous injection or bedaquiline at 25 mg/kg and/or PA-824 at 50 mg/kg by oral gavage. Isoniazid at 25 mg/kg, rifampicin at 10 mg/kg and pyrazinamide at 150 mg/kg were given by oral gavage. Initiation of treatment controls are the number of log_10_ cfu in lungs at the start of treatment, 12 days post-aerosol infection. BDQ, bedaquiline; INH, isoniazid; PZA, pyrazinamide; RIF, rifampicin; NS, not significant; ***P <* *0.001.

As an injectable drug with potential to treat MDR TB, the activity of 1599 alone and in combination with anti-MDR TB agents was first studied using the BALB/c high-dose aerosol model. In this model, bacterial cfu in the lungs of mice increased between day 1 and day 12 (start of treatment) (Table 1). By day 16 post-aerosol exposure, all untreated mice had become moribund, showing clinical signs of infection (i.e. lethargy, trembling and weight loss) and were humanely euthanized. All other mice survived to the end of the study. As tested, PA-824, 1599 and linezolid monotherapies reduced lung burdens by 1.80, 2.15 and 2.18 log, respectively (*P *<* *0.001). Bedaquiline exhibited the best overall efficacy when employed in monotherapy, with a 4.27 log reduction in lung bacterial burdens relative to the start-of-treatment control group. Comparing the log_10_ cfu reductions in lungs, the combination of 1599 with linezolid, bedaquiline or PA-824 did not result in any net overall improvement in efficacy relative to the most efficacious of the paired agents when dosed in monotherapy (*P *>* *0.05), but 1599 was not antagonistic in any of these cases either (Table 1). In contrast, co-administration of PA-824 with bedaquiline resulted in a significant decrease in efficacy compared with bedaquiline given alone (*P *<* *0.001; Figure 1 and Table 1), which has been reported previously.^16^^,^^19^ Appreciable *in vivo* benefit was observed when 1599 was paired with bedaquiline/PA-824. This combination resulted in a significant overall improvement in lung activity by >1 log relative to the bedaquiline/PA-824 two-drug combination (*P *<* *0.001), but was not sufficient to overcome the observed antagonism of PA-824 on bedaquiline *in vivo* (Figure 1 and Table 1). No net improvement was seen when 1599 was combined with bedaquiline/linezolid, whereas the inclusion of 1599 with bedaquiline/pyrazinamide resulted in an additional ∼0.66 log of killing relative to the bedaquiline/pyrazinamide comparator (*P *<* *0.001; Table 1). Each of the 1599-containing three-drug combinations showed significant improvement over the first-line regimen of isoniazid/rifampicin/pyrazinamide in this short-term study (*P *<* *0.05; Table 1). However, the best overall *in vivo* activity was seen with 1599/bedaquiline/pyrazinamide, which resulted in an additional 2.73 log net improvement over isoniazid/rifampicin/pyrazinamide with 3 weeks of treatment (*P *<* *0.001; Table 1).

**Table 1:** Combination of 1599 with second-line and novel drugs under development in BALB/c mice following high-dose aerosol infection with *M. tuberculosis* Erdman-Lux

	Therapy administered			In combination with 1599		
Drug (dose)^a^	log_10_ lung cfu ± SEM	log reduction	number of mice in each group	log_10_ lung cfu ± SEM	log reduction	number of mice in each group
D1, untreated controls	4.03 ± 0.04	NA	3			
D12, untreated controls	7.58 ± 0.05	NA	6			
D33, 3 weeks of 5 of 7 day treatments (M-F)^b^						
1599 (200)	5.43 ± 0.02	2.15	6			
rifampicin (10)	6.50 ± 0.16	1.08	5	4.61 ± 0.12	2.97	6
pyrazinamide (150)	6.60 ± 0.15	0.98	5	ND		
bedaquiline (25)	3.31 ± 0.06	4.27	6	3.40 ± 0.04	4.18	6
PA-824 (50)	5.78 ± 0.03	1.80	6	5.37 ± 0.04	2.21	6
linezolid (100)	5.40 ± 0.10	2.18	6	5.12 ± 0.05	2.46	6
bedaquiline/linezolid	3.65 ± 0.05	3.93	6	3.82 ± 0.16	3.76	6
bedaquiline/pyrazinamide	3.07 ± 0.07	4.51	6	2.41 ± 0.11	5.17	6
bedaquiline/PA-824	5.57 ± 0.07	2.21	6	4.63 ± 0.13	2.95	6
isoniazid/rifampicin/pyrazinamide	5.14 ± 0.03	2.44	6			

NA, not applicable; ND, not done.

D1, D12 and D33 are 1, 12 and 33 days post-aerosol infection.

Drug treatment started on day 12.

a Drug dose in milligrams per kilogram of body weight.

b Drug treatments Monday to Friday.

### Bactericidal activity of 1599 with first-line TB drugs in BALB/c mice

**Figure 2:**
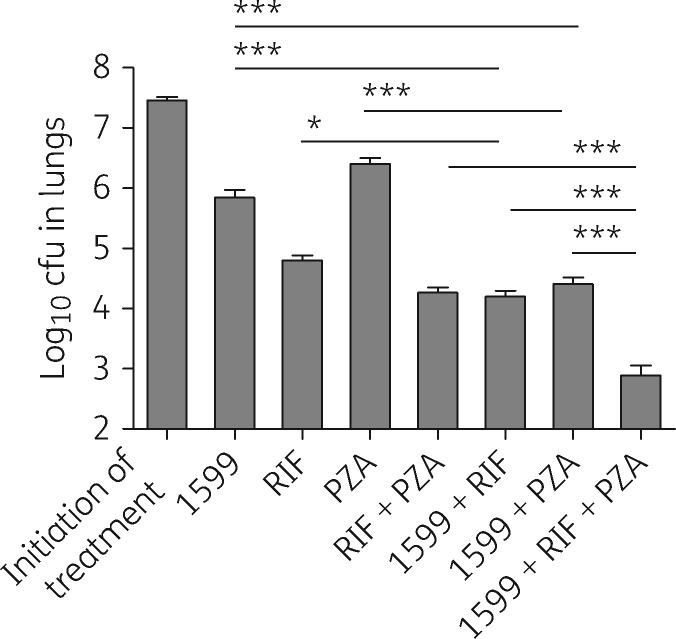
1599 combination studies with rifampicin and pyrazinamide in BALB/c mice following high-dose aerosol infection with *M. tuberculosis* Erdman. Bars represent mean log_10_ cfu values ±SEM from the lungs of individual BALB/c mice. Mice were treated for 4 weeks with the following drugs, alone or in various combinations: 1599 at 200 mg/kg by subcutaneous injection, or rifampicin at 10 mg/kg, and pyrazinamide at 150 mg/kg by oral gavage. Initiation of treatment controls are the number of log_10_ cfu in lungs at the start of treatment, 12 days post-aerosol infection. PZA, pyrazinamide; RIF, rifampicin; *P <* *0.05; ***P <* *0.001.

To evaluate the potential *in vivo* benefit of combining 1599 with first-line TB drugs, the activities of the four front-line agents alone or in two- and three-drug combinations with 1599 were compared using the BALB/c high-dose aerosol model. Comparing the log_10_ cfu reductions in lungs, the combination of 1599 with rifampicin, pyrazinamide or ethambutol significantly improved treatment outcome over that observed with either agent when given alone (*P* < 0.001; Table 2). Thus, these results revealed a net overall improvement in efficacy when 1599 was administered with the key first-line anti-TB agents, rifampicin, pyrazinamide or ethambutol. Moxifloxacin, studied in Phase III clinical trials for treatment of drug-susceptible TB,^20^ also showed better *in vivo* activity when co-administered with 1599 (*P* = 0.01; Table 2). In contrast, isoniazid proved significantly less effective when co-administered with 1599, indicating some antagonism (*P* < 0.01; Table 2). In a three-drug combination, 1599 proved highly effective when paired with rifampicin/pyrazinamide, reducing lung burdens from treatment start by >4.5 log, which is significantly better than that observed with rifampicin/pyrazinamide, 1599/rifampicin or 1599/pyrazinamide (*P* < 0.001; Figure 2 and Table 2). Similar results were observed with a second lead spectinamide, 1810, which showed that the effect is class-specific (Table 2).

**Table 2:** Combination of spectinamides with first-line antitubercular agents in BALB/c mice following high-dose aerosol infection with *M. tuberculosis* Erdman

	Therapy administered			In combination with 1599			In combination with 1810		
Drug (dose)^a^	log_10_ lung cfu ± SEM	log reduction	number of mice in each group	log_10_ lung cfu ± SEM	log reduction	number of mice in each group	log_10_ lung cfu ± SEM	log reduction	number of mice in each group
D1, untreated controls	4.71 ± 0.06	NA	3						
D12, untreated controls	7.46 ± 0.06	NA	6						
D40, 4 weeks of 5 of 7 day treatments (M-F)^b^									
1599 (200)	5.84 ± 0.13	1.62	5						
1810 (200)	5.69 ± 0.17	1.77	6						
rifampicin (10)	4.80 ± 0.08	2.66	6	4.20 ± 0.10	3.26	6			
pyrazinamide (150)	6.40 ± 0.10	1.06	6	4.41 ± 0.11	3.05	6			
isoniazid (25)	4.67 ± 0.06	2.79	6	5.32 ± 0.10	2.14	6			
ethambutol (100)	6.12 ± 0.12	1.34	6	5.02 ± 0.05	2.44	6			
moxifloxacin (100)	4.65 ± 0.12	2.81	6	4.07 ± 0.05	3.39	6			
rifampicin/pyrazinamide	4.26 ± 0.09	3.20	6	2.89 ± 0.16	4.57	5	3.22 ± 0.11	4.24	6

NA, not applicable.

D1, D12 and D40 are 1, 12 and 40 days post-aerosol infection.

Drug treatment started on day 12.

a Drug dose in milligrams per kilogram of body weight.

b Drug treatments Monday to Friday.

**Figure 3:**
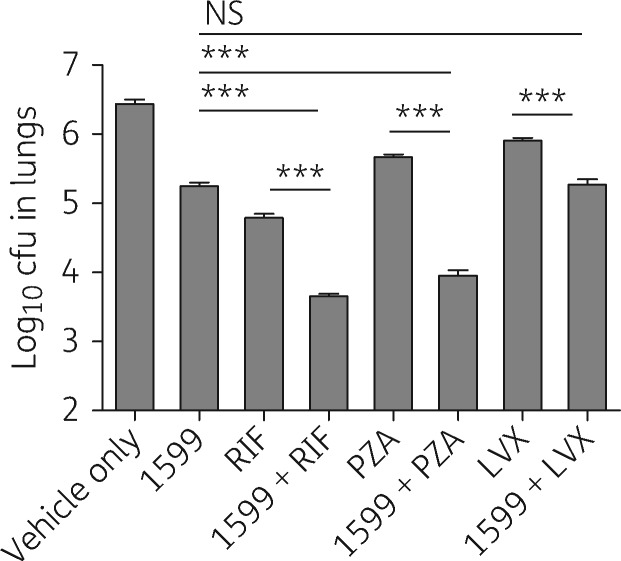
1599 combination studies with rifampicin, pyrazinamide or levofloxacin in BALB/c mice chronically infected with *M. tuberculosis* Erdman-Lux. Mice were treated for 4 weeks with the following drugs, alone or in various combinations: 1599 at 200 mg/kg by subcutaneous injection, rifampicin at 10 mg/kg, pyrazinamide at 150 mg/kg or levofloxacin at 100 mg/kg by oral gavage. The vehicle-only group received Plasma-Lyte by subcutaneous injection. LVX, levofloxacin; PZA, pyrazinamide; RIF, rifampicin; NS, not significant; ***P <* *0.001.

A similar improvement in efficacy was observed when rifampicin or pyrazinamide was paired with 1599 in chronically infected BALB/c mice infected by low-dose aerosol.^14^^,^^21^ Infection in this manner results in a low initial bacterial burden, which plateaus and stabilizes after the onset of acquired immunity.^7^ Four weeks of treatment with rifampicin, pyrazinamide or 1599 in this model reduced lung burdens by 1.65, 0.77 and 1.19 log, respectively (*P* < 0.001; Table S1). Appreciably better efficacy was observed in mice treated with 1599/rifampicin (additive) or 1599/pyrazinamide (synergistic) compared with rifampicin or pyrazinamide alone in the chronic infection model (*P* < 0.001; Figure 3 and Table S1). In contrast, co-administration of 1599 with levofloxacin, a second fluoroquinolone, resulted in no further reduction in lung burdens relative to that observed with 1599 alone (*P* > 0.05; Figure 3 and Table S1).

### Activity of 1599 alone and in combination with rifampicin and/or pyrazinamide in the C3HeB/FeJ mouse model

**Figure 4:**
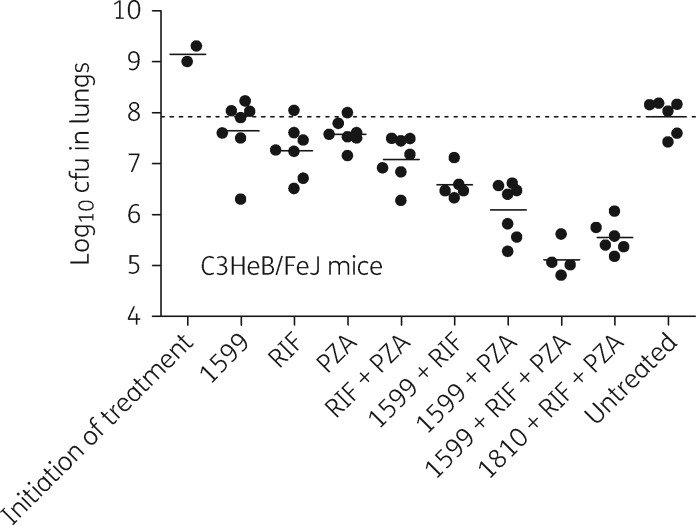
1599 and 1810 combination studies with rifampicin and pyrazinamide in C3HeB/FeJ mice experimentally infected with *M. tuberculosis* Erdman. Plots represent the log_10_ cfu values from the lungs of individual mice. The mean log_10_ cfu value is represented by a horizontal line. The broken line represents the mean log_10_ cfu value for the untreated control. Fifty-six days following low-dose aerosol infection, mice were treated for 4 weeks with the following drugs, alone or in various combinations: 1599 or 1810 at 200 mg/kg by subcutaneous injection, or rifampicin at 10 mg/kg, and pyrazinamide at 150 mg/kg by oral gavage. The untreated control group received no treatment. PZA, pyrazinamide; RIF, rifampicin.

Because of the striking improvements in *in vivo* activity observed for 1599, rifampicin and/or pyrazinamide in the acute and chronic BALB/c mouse models, we sought to evaluate these combinations in C3HeB/FeJ mice infected by low-dose aerosol with *M. tuberculosis* Erdman. Early mortality in a subset (roughly 20%–40%) of infected, but untreated, C3HeB/FeJ mice is a hallmark of productive infection in this model.^6^ Here, roughly 60% of the infected mice became moribund between days 28 and 45 post-aerosol exposure due to TB infection and were humanely euthanized prior to the start of treatment. Histological examination of lung tissue from six untreated control mice that had survived this early mortality phase to day 56 post-aerosol exposure (start of treatment) revealed the presence of the three characteristic lung lesion types (not shown).^6^ At this time bacterial loads in the lungs of additional untreated mice had reached 9.09 log_10_ cfu. These data indicate that a productive infection characterized by high bacterial titres and advanced caseous necrotic pulmonary lesions was established. Such lesions better resemble the heterogeneous lung pathology seen in humans and are not similarly observed in conventional mouse models.^22^ Four weeks of treatment with 1599, rifampicin or pyrazinamide alone in C3HeB/FeJ mice resulted in only modest, but not significant, reductions in bacterial loads in lungs of C3HeB/FeJ mice (Figure 4 and Table S2). Heterogeneity in treatment response was also observed and is characteristic for treatment outcome in this model (Figure 4).^5^^,^^6^^,^^23–27^ Combination of 1599 with pyrazinamide significantly outperformed treatments with either 1599 or pyrazinamide alone (*P* < 0.001); co-administration of rifampicin with 1599 also resulted in statistically significant net improvement in efficacy relative to that observed with 1599 monotherapy alone (*P* = 0.005; Figure 4 and Table S2). Strikingly, 1599 was synergistic when combined with both rifampicin and pyrazinamide (1599/rifampicin/pyrazinamide), resulting in a 2.81 log_10_ reduction versus the untreated control (*P* < 0.001), and showing significant improvement over that of rifampicin/pyrazinamide (*P* < 0.001), 1599/rifampicin (*P* < 0.001) or 1599/pyrazinamide (*P* = 0.026; Figure 4 and Table S2). This was reproducible in a second independent experiment (Table S3) and class specific since it was similarly observed with a second lead spectinamide, 1810 (Figure 4 and Table S2). Thus, the *in vivo* synergistic effect of spectinamides (i.e. 1599 and 1810) with rifampicin and pyrazinamide was conserved from conventional mice to the more advanced C3HeB/FeJmousemodel (compare Figure 2 and Figure 4).

### In vitro assessment of synergy

An interesting result of this study was the lack of correlation between the *in vitro* synergy and the *in vivo* synergy. It would be a rational expectation that the *in vivo* synergy observed when 1599 was paired with rifampicin, pyrazinamide or bedaquiline would be reflective of the *in vitro* mode of action of each agent. However, when tested in conventional MIC *in vitro* chequerboard assays, 1599 in combination with rifampicin, pyrazinamide or bedaquiline did not reveal any specific synergies, although none of these interactions was antagonistic either (Table S4). Some synergy (FICI 0.13–0.25) was observed when 1599 was paired with clofazimine or rifapentine *in vitro* (Table S4); however, these agents have yet to be evaluated for activity *in vivo* when combined with a spectinamide.

## Discussion

In the studies presented here, we report the *in vivo* benefit of combining 1599 with existing and novel anti-TB therapies in multiple short-course murine infection models as a means to assess potential mechanistic synergy partners. We demonstrate: (i) mild antagonism with isoniazid, a drug that has been reported before to be weakly antagonistic with rifampicin and pyrazinamide in certain mouse models,^28^^,^^29^ but no interference or adverse effects when 1599 is administered in combination with other agents tested here; (ii) 1599 in combination with rifampicin and/or pyrazinamide was additive or synergistic in three mouse models evaluated (the acute and chronic BALB/c as well as the C3HeB/FeJ model); and (iii) the most effective combination tested was the three-drug regimen combining 1599 with bedaquiline/pyrazinamide. Importantly, none of these synergistic interactions was predicted from *in vitro* MIC chequerboard assays, suggesting that current *in vitro* methodologies cannot replace conventional *in vivo* drug combination studies for TB drug development. Additionally, while appreciable *in vivo* benefit was observed when moxifloxacin was paired with 1599 in the high-dose aerosol model, this was not similarly the case for a second fluoroquinolone, levofloxacin, when tested in chronically infected mice. Whether this reflects differences in these two models or the superior bactericidal activity of moxifloxacin^30^ remains to be determined.

The spectinamide 1599 was highly effective *in vivo* when paired with rifampicin. Mechanistically, both drugs effect direct inhibition of protein synthesis, albeit at different drug targets (i.e. rifampicin at the level of mRNA synthesis and 1599 as an inhibitor of ribosome translocation). Appreciable synergy was also observed in a previously published study where 1599 was paired with another protein synthesis inhibitor, clarithromycin.^4^ It is therefore plausible to suggest that inhibition of two different steps within the same pathway could result in a complete shutdown of protein synthesis. Targeting protein synthesis in multiple ways is, however, not a guarantee of enhanced *in vivo* activity, as this was not observed when 1599 was paired with linezolid, which acts at the initiation phase of protein synthesis.^31^ No improvement in potency was observed when rifampicin was combined with 1599 in *in vitro* MIC chequerboard assays either. Alternative explanations for the improved potency of this combination may relate to increased retention or uptake of rifampicin into the bacterium in the presence of 1599 or killing of rifampicin-tolerant subpopulations by 1599. This could be through an alteration of efflux since the intrinsic efflux pump (Rv1258c) avoided by the spectinamides has been shown to contribute to *in vivo* growth and also macrophage-induced rifampicin tolerance.^32^ Finally, changes in pharmacokinetic properties upon combining drugs, which were not assessed in these early *in vivo* studies, will be investigated further.

Pyrazinamide has previously been shown to be synergistic with several new TB agents in development.^33–36^ When combined with 1599 herein, it also showed promising synergism, especially in the C3HeB/FeJ model. In our studies, pyrazinamide showed a range in efficacy dependent on the mouse infection model utilized. Reduction in bacterial loads was highest in the acute BALB/c mouse model, modest in the chronic model and lower in C3HeB/FeJ mice. These results are consistent with earlier published data on pyrazinamide in C3HeB/FeJ mice, where the limited potency observed was thought to arise from the lack of a sufficiently acidic environment to support the antimicrobial activity of pyrazinamide within the neutral to slightly basic caseum.^22^^,^^25^^,^^37^^,^^38^ Pyrazinamide has been shown in earlier work to show some activity at neutral pH under certain *in vitro* conditions, many of which are in fact present in necrotic lesions in C3HeB/FeJ mice *in vivo* (e.g. hypoxia^8^ and nutrient starvation^39^). A recent study provided more evidence to support this by demonstrating that susceptibility to pyrazinamide and its biologically active metabolite (pyrazinoic acid) *in vitro* is independent of environmental pH under certain conditions.^40^ Consequently, the limited activity of pyrazinamide when tested alone in C3HeB/FeJ mice might only be explained by contributions of multiple micro-environmental stressors present in necrotic lesions affecting the metabolic state of bacteria in a profound and specific way, and not by pH alone. Pyrazinamide also exhibits activity with bedaquiline in C3HeB/FeJ mice,^38^ suggesting that the pyrazinamide potentiating effect may not be specific to the spectinamides, *per se*. These observations suggest that the synergistic effects of pyrazinamide with companion drugs and the antibacterial activity observed when used as a single drug may be separable mechanisms. Cooperative mechanisms of action,^16^^,^^35^^,^^41^ different target subpopulations of metabolically distinct bacteria or pharmacokinetic effects may also contribute to the observed efficacy when used in combination.

Of all drug regimens evaluated, the most remarkable result in terms of synergy was obtained with the combination of rifampicin, pyrazinamide and a spectinamide (i.e. 1599 or 1810) in C3HeB/FeJ mice. The observed synergy was especially striking given the limited overall activity of each drug alone in this mouse model. The effect is clearly dependent on the addition of a spectinamide since the combined efficacy of all three drugs is significantly higher than the rifampicin/pyrazinamide combination tested without a spectinamide. C3HeB/FeJ mice are known to exhibit advanced lung pathology upon *M. tuberculosis* infection.^42^ Compared with that observed in more conventional mouse models, the presence of advanced, hypoxic, caseating lung lesions and the higher ratio of extracellular to intracellular bacilli in C3HeB/FeJ mice has been shown to have a profound effect on treatment outcome with multiple chemotherapeutic agents,^6^^,^^23–27^ including rifampicin and pyrazinamide.^5^^,^^25^^,^^37^ Our studies therefore suggest that the combination of 1599 with rifampicin and pyrazinamide is able to significantly enhance drug efficacy of both rifampicin and/or pyrazinamide in this advanced disease model during a 4 week treatment regimen, which warrants further testing in long-term trials.

In conclusion, the data presented in this study support the further study of the spectinamides as combination partners with existing and experimental classes of anti-TB agents. While synergy between two antibiotics is often demonstrated *in vitro*, such a demonstration *in vivo* in complex disease infection models, where the sum of the individual drug response is lower than that observed for the combination, is a rarity. It is not yet known whether the improvements in *in vivo* activity observed herein result from mechanistic interactions or from alterations in drug exposure, drug uptake or retention. Further studies are clearly warranted to better understand the molecular basis for these interactions and to explore the sterilizing activity of spectinamide/rifampicin/pyrazinamide and spectinamide/bedaquiline/pyrazinamide combinations in mouse relapse models. It is our hope that detailed exploration of the molecular mechanism of synergy between the spectinamides, pyrazinamide and rifampicin, may aid the discovery of novel approaches to treat TB and to shorten therapy in humans.

## Supplementary Material

Supplementary Data
