# Supplementary material for: Spectinamides are effective partner agents for the treatment of tuberculosis in multiple mouse infection models
Source: J Antimicrob Chemother. 2016 Dec 13;72(3):770–7. doi: 10.1093/jac/dkw467 (PMC5400088; doi:10.1093/jac/dkw467)
Supplement: Supplementary Data [file dkw467_Supplementary_Data.zip › Supplementary_Data.doc]

**Supplementary data**

**Figure S1.** Structures of spectinomycin and the semisynthetic spectinamide derivatives 1599 and 1810.

**Table S1.** Combination of 1599 with first-line antitubercular agents in a BALB/c chronic mouse infection model following low-dose aerosol infection with *M. tuberculosis* Erdman-Lux.

| **Drug (dose)^a^** | **Therapy administered** | | | **In combination with 1599** | | |
| --- | --- | --- | --- | --- | --- | --- |
|  | **Log_10_ Lung CFU ± SEM** | **Log Reduction** | **n** | **Log_10_ Lung CFU ± SEM** | **Log Reduction** | **n** |
| D1, untreated controls | 2.56 ± 0.06 | na | 3 |  |  |  |
| D41, untreated controls | 7.04 ± 0.06 | na | 6 |  |  |  |
|  |  |  |  |  |  |  |
| D69, 4 wks of 5 of 7 day treatments (M-F) | | |  |  |  |  |
| Vehicle only | 6.44 ± 0.06 | na | 6 |  |  |  |
|  |  |  |  |  |  |  |
| 1599 (200) | 5.25 ± 0.05 | 1.19 | 5 |  |  |  |
| RIF (10) | 4.79 ± 0.06 | 1.65 | 6 | 3.65 ± 0.04 | 2.79 | 6 |
| PZA (150) | 5.67 ± 0.04 | 0.77 | 6 | 3.95 ± 0.08 | 2.49 | 5 |
| LVX (100) | 5.91 ± 0.04 | 0.53 | 6 | 5.27 ± 0.08 | 1.17 | 4 |

^a^Drug dose in milligrams per kilogram of body weight. D1, D41, and D69 are 1, 41, and 69 days post-aerosol infection. Drug-treatment started on day 41. Abbreviations. CFU, colony forming units; SEM, standard error of the mean; na, not applicable; RIF, rifampicin; PZA, pyrazinamide; LVX, levofloxacin.

**Table S2**. Combination of 1599 with first-line antitubercular agents in a C3HeB/FeJ mouse infection model following low-dose aerosol infection with *M. tuberculosis* Erdman.

| **Drug (dose)^a^** | **Therapy administered** | | | **In combination with 1599** | | | **In combination with 1810** | | |
| --- | --- | --- | --- | --- | --- | --- | --- | --- | --- |
|  | **Log_10_ Lung CFU ± SEM** | **Log Reduction** | **n** | **Log_10_ Lung CFU ± SEM** | **Log Reduction** | **n** | **Log_10_ Lung CFU ± SEM** | **Log Reduction** | **n** |
| D1, untreated controls | 1.80 ± 0.25 | na | 3 |  |  |  |  |  |  |
| D56, untreated controls | 9.09 ± 0.13 | na | 2 |  |  |  |  |  |  |
|  |  |  |  |  |  |  |  |  |  |
| D84, 4 wks of 5 of 7 day treatments (M-F) | | |  |  |  |  |  |  |  |
| Untreated | 7.92 ± 0.14 | na | 7 |  |  |  |  |  |  |
|  |  |  |  |  |  |  |  |  |  |
| 1599 (200) | 7.65 ± 0.25 | 0.27 | 7 |  |  |  |  |  |  |
| RIF (10) | 7.26 ± 0.20 | 0.66 | 7 | 6.58 ± 0.14 | 1.34 | 5 |  |  |  |
| PZA (150) | 7.59 ± 0.10 | 0.33 | 7 | 6.09 ± 0.21 | 1.83 | 5 |  |  |  |
| RIF + PZA | 7.08 ± 0.17 | 0.84 | 7 | 5.11 ± 0.17 | 2.81 | 4 | 5.55 ± 0.13 | 2.37 | 6 |

^a^Drug dose in milligrams per kilogram of body weight. D1, D56, and D84 are 1, 56, and 84 days post-aerosol infection. Drug-treatment started on day 56. Abbreviations. CFU, colony forming units; SEM, standard error of the mean; na, not applicable; RIF, rifampicin; PZA, pyrazinamide.

**Table S3.** Combination of 1599 with first-line antitubercular agents in a C3HeB/FeJ mouse infection model following low-dose aerosol infection with *M. tuberculosis* Erdman – second study.

|  | **Therapy administered** | | | **In combination with 1599** | | |
| --- | --- | --- | --- | --- | --- | --- |
| **Drug (dose)^a^** | **Log_10_ Lung CFU ± SEM** | **Log Reduction** | **n** | **Log_10_ Lung CFU ± SEM** | **Log Reduction** | **n** |
| D1, untreated controls | 2.18 ± 0.45 | na | 3 |  |  |  |
| D56, untreated controls | 8.82 ± 0.22 | na | 6 |  |  |  |
|  |  |  |  |  |  |  |
| D84, 4 wks of 5 of 7 day treatments (M-F) | | |  |  |  |  |
| Vehicle only | 8.19 ± 0.69 | na | 4 |  |  |  |
|  |  |  |  |  |  |  |
| 1599 (200) | 7.91 ± 0.26 | 0.28 | 5 |  |  |  |
| RIF + PZA | 7.25 ± 0.21 | 0.93 | 7 | 4.86 ± 0.16 | 3.33 | 6 |

^a^Drug dose in milligrams per kilogram of body weight. D1, D56, and D84 are 1, 56, and 84 days post-aerosol infection. Drug-treatment started on day 56. Abbreviations. CFU, colony forming units; SEM, standard error of the mean; na, not applicable; RIF, rifampicin.

**Table S4.** Growth inhibition of *M. tuberculosis* H37Rv using an *in vitro* MIC checkerboard approach.

| **Test agent** | **Drug Class** | **^a^TB therapy** | **MIC Alone (mg/L)** | **^b^FICI with 1599** | **Type of interaction** |
| --- | --- | --- | --- | --- | --- |
| 1599 | Spectinamide | Experimental | 0.8-1.6 | na | na |
|  |  |  |  |  |  |
| Rifampicin | Rifamycins | First-line | 0.02-0.2 | 0.5-1.0 | indifference |
| Rifabutin | Rifamycins | Non-traditional | 0.03 | 0.8 | indifference |
| Rifapentine | Rifamycins | Second-line | 0.1-0.5 | 0.16-0.25 | **synergy** |
| Kanamycin | Aminoglycosides | Second-line | 3.1 | 1.0 | indifference |
| Amikacin | Aminoglycosides | Non-traditional | 0.78 | 2.0 | indifference |
| Ofloxacin | Fluoroquinolones | Second-line | 3.1 | 2.0 | indifference |
| Levofloxacin | Fluoroquinolones | Second-line | 12.5 | 2.0 | indifference |
| Moxifloxacin | Fluoroquinolones | Non-traditional | 0.2 | 2.0 | indifference |
| PA824 | Nitroimidazoles | Non-traditional | 0.78 | 1.0 | indifference |
| Isoniazid | Antimycobacterial | First-line | 0.05 | 2.0 | indifference |
| Pyrazinamide (pH 7.0) | Antimycobacterial | First-line | >200 | 1.0 | indifference |
| Pyrazinamide (pH 5.5) | Antimycobacterial | First-line | 100 | 1.0 | indifference |
| Ethambutol | Antimycobacterial | First-line | 1.6-6.3 | 2.0 | indifference |
| Clofazimine | Antimycobacterial | Second-line | 1.6 | 0.13 | **synergy** |
| Ethionamine | Antimycobacterial | Second-line | 25 | 1.0 | indifference |
| Cycloserine | Antimycobacterial | Second-line | 1.6 - 6.3 | 0.5-2.0 | indifference |
| Bedaquiline | Antimycobacterial | Non-traditional | 0.4 | 1.0 | indifference |
| SQ109 | Antimycobacterial | Non-traditional | 1.56 | 1.0 | indifference |

^a^Use in tuberculosis chemotherapy

^b^Fractional inhibitory concentration index (FICI) scores were calculated using the formula [MIC drug B in presence of Drug A] / [MIC of drug B] + [MIC of drug A in the presence of drug B] / [MIC of drug A]. FICI scores were interpreted as follows: synergy (< 0.5), indifference (> 0.5 – 4.0), or antagonism (> 4.0)
